# Overnight Sleep Staging Using Chest-Worn Accelerometry

**DOI:** 10.3390/s24175717

**Published:** 2024-09-02

**Authors:** Fons Schipper, Angela Grassi, Marco Ross, Andreas Cerny, Peter Anderer, Lieke Hermans, Fokke van Meulen, Mickey Leentjens, Emily Schoustra, Pien Bosschieter, Ruud J. G. van Sloun, Sebastiaan Overeem, Pedro Fonseca

**Affiliations:** 1 Department of Electrical Engineering, Eindhoven University of Technology, 5612 AP Eindhoven, The Netherlands; m.ross@tue.nl (M.R.); l.w.a.hermans@tue.nl (L.H.); meulenf@kempenhaeghe.nl (F.v.M.); p.f.n.bosschieter@tue.nl (P.B.); r.j.g.v.sloun@tue.nl (R.J.G.v.S.); s.overeem@tue.nl (S.O.); pedro.fonseca@philips.com (P.F.); 2 Philips Sleep and Respiratory Care, 5656 AE Eindhoven, The Netherlands; angela.grassi@philips.com; 3 The Siesta Group, 1210 Vienna, Austria; peter.anderer@thesiestagroup.com; 4 FH Technikum Wien, 1200 Wien, Austria; cerny@technikum-wien.at; 5 Center for Sleep Medicine Kempenhaeghe, 5591 VE Heeze, The Netherlands; 6 Department of Otorhinolaryngology, Head and Neck Surgery OLVG West, 1061 AE Amsterdam, The Netherlands; m.leentjens@olvg.nl (M.L.); e.schoustra@olvg.nl (E.S.)

**Keywords:** hypnogram, sleep staging, sleep metrics, accelerometer, artificial intelligence

## Abstract

Overnight sleep staging is an important part of the diagnosis of various sleep disorders. Polysomnography is the gold standard for sleep staging, but less-obtrusive sensing modalities are of emerging interest. Here, we developed and validated an algorithm to perform “proxy” sleep staging using cardiac and respiratory signals derived from a chest-worn accelerometer. We collected data in two sleep centers, using a chest-worn accelerometer in combination with full PSG. A total of 323 participants were analyzed, aged 13–83 years, with BMI 18–47 kg/m^2^. We derived cardiac and respiratory features from the accelerometer and then applied a previously developed method for automatic cardio-respiratory sleep staging. We compared the estimated sleep stages against those derived from PSG and determined performance. Epoch-by-epoch agreement with four-class scoring (Wake, REM, N1+N2, N3) reached a Cohen’s kappa coefficient of agreement of 0.68 and an accuracy of 80.8%. For Wake vs. Sleep classification, an accuracy of 93.3% was obtained, with a sensitivity of 78.7% and a specificity of 96.6%. We showed that cardiorespiratory signals obtained from a chest-worn accelerometer can be used to estimate sleep stages among a population that is diverse in age, BMI, and prevalence of sleep disorders. This opens up the path towards various clinical applications in sleep medicine.

## 1. Introduction

Assessing overnight sleep structure is a cornerstone of the somnological diagnostic process and is an important step in the diagnosis of various sleep disorders. The gold standard for the scoring of sleep stages uses the rules of the American Academy of Sleep Medicine (AASM) and is based on neurological signals measured during polysomnography (PSG), specifically electroencephalography (EEG), electrooculography (EOG) and electromyography (EMG) [1]. Sleep stages encompass “light sleep” (stage N1 and N2), “deep sleep” (slow wave sleep, stage N3) and REM sleep. The occurrence of sleep stages overnight is typically plotted in a hypnogram to visualize sleep structure. Furthermore, derived metrics such as total sleep time (TST) or sleep efficiency are often some of the diagnostic indicators describing the burden or effect of a sleep disorder. One important example is the Apnea Hypopnea Index (AHI) as a severity estimator for obstructive sleep apnea (OSA). The AHI is defined as the number of apnea and hypopnea events per hour of sleep. As such, estimation of total sleep time is crucial for a proper determination of the AHI. The absence of sleep staging (e.g., in reduced montage polygraphy) forces the use of total recording time instead of TST and leads to an associated underestimation of the AHI [2]. 

PSG may be conducted in a clinic or at home but is often restricted to a single night because of its obtrusiveness and associated handling costs. PSG is therefore impractical for the long-term monitoring of sleep at home. Furthermore, to determine the efficacy of a sleep-related therapy, prolonged measurements are required as well. To enable such measurements, alternatives to PSG have been studied over the past decade. One approach uses EEG measurements with a reduced number of electrodes, which can be self-attached [3,4]. In other, increasingly popular approaches, sleep staging is performed without EEG, but with the measurement of cardiac and/or respiratory activity instead. These exploit the increasingly well-understood link between autonomic nervous system activity and sleep stages [5,6,7]. For example, heart rate decreases during deeper non-REM sleep stages (N2-N3), while REM is a period of autonomic instability, with heart rate variability reflecting the constant change in balance between sympathetic and parasympathetic tone. Comparably, respiration becomes more regular in non-REM stages and less regular during REM sleep [8,9,10]. 

With advances in machine learning, it has become possible to exploit these variations in cardiac and respiratory signals for the estimation of sleep stages. Cardiorespiratory signals thus act as a “surrogate”, as opposed to the neurological signals that are the formal foundation for sleep staging [11]. In a recent review, researchers showed that approaches based on the simultaneous use of cardiac and respiratory signals tend to achieve the most accurate results [12]. Note that, as differences between sleep stages may be less apparent in cardiorespiratory signals, classification is often performed with a reduced number of stages, for example Wake/REM/Non-REM (3-class), or Wake/REM/N1+N2/N3 (4-class). The CardioRespiratory Sleep Staging (CReSS) algorithm uses both cardiac and respiratory signals to perform 4-class staging. CReSS was validated using 296 overnight PSG recordings, and a substantial agreement was found between sleep stages derived from the AASM-recommended signals for sleep staging (EEG, EOG, EMG) and those derived from only the cardiorespiratory signals in the PSG [13].

Recent advances in wearable cardiorespiratory sensors, for example based on photoplethysmography (PPG), but also low-power and low-noise accelerometers, enable new approaches to longitudinal and minimally obtrusive measurement of sleep in general and sleep staging in particular [14]. These developments have been the driver behind a plethora of sleep trackers that, to some degree, allow for the assessment of sleep–wake patterns in healthy individuals [15,16]. Further developments in artificial intelligence, in combination with the availability of clinical datasets that combine PSG with new sensors, have led to substantial performance improvements, as well as in simplified sleep staging tasks. In one study, for example, the use of heart rate variability derived from wrist PPG, in combination with actigraphy derived from wrist accelerometry, enabled sleep staging in a clinical population [17].

Another interesting sensing modality in this setting is chest-worn accelerometry. These sensors are typically very small, consume little power, and can be integrated in devices that are worn on the chest anyway, such as monitoring patches and therapy devices for positional obstructive sleep apnea [18,19]. In addition, such a device only requires a mechanical coupling with the chest at a single point, without the need for direct skin contact, so that it can be worn in or over clothing and does not depend on galvanic or optical contact properties.

Recent studies have shown that cardio-respiratory metrics can be accurately and robustly derived from a chest-worn accelerometer. By measuring small changes in the orientation of a chest-worn accelerometer and processing these with a deep neural network, respiratory effort can be estimated [20]. Cardiac activity can be measured by a method that uses tiny vibrations in the chest wall, in combination with maximum a posteriori estimation to localize heartbeats and to estimate inter-beat intervals (IBIs) [21]. 

The objective of this study is to investigate whether sleep stages and associated sleep metrics can be accurately estimated from a chest-worn accelerometer, leveraging the recently developed methods for estimation of cardiac and respiratory activity from this sensor, in combination with established methods for cardiorespiratory sleep staging [13].

## 2. Materials and Methods

### 2.1. Data Collection

Data were collected in two sleep centers from participants undergoing full overnight PSG, as part of a routine investigation because of a suspected sleep disorder. Simultaneous with the PSG, a triaxial accelerometer device (ADXL355, Analog Devices, Wilmington, MA, USA) was mounted in a housing measuring 62 × 48 × 11 mm on the thoracic respiratory band, approximately 50 mm left of the sternum. The three channels of the accelerometer (x, y, and z) were sampled and recorded at 250 Hz.

The first center (KH) was the Sleep Medicine Center Kempenhaeghe in Heeze, The Netherlands. Data collection was part of the SOMNIA project, reviewed by the Medical Ethical Committee of the Maxima Medical Center (Eindhoven, The Netherlands. File no: N16.074) [22]. The second center (OLVG) was the Sleep Center of the OLVG Hospital in Amsterdam, the Netherlands. The study protocol was reviewed and approved by the local scientific advice committee of OLVG (Amsterdam, The Netherlands. File no: WO 20.134). Both protocols were approved by the Internal Committee of Biomedical Experiments of Philips Research. Recruitment was among participants scheduled for overnight PSG as part of the standard diagnostic process. Participants undergoing CPAP titration, with intellectual disabilities, or with an earlier diagnosis of atrial fibrillation were excluded. All participants provided consent prior to study initiation. In OLVG, 10% of the recordings were made in the clinic, with in-lab PSG, while the remaining 90% were made at home, with ambulatory PSG. All sensors, including the accelerometer device, were mounted by the clinical staff in the hospital, also for the ambulatory recordings.

### 2.2. Data Preprocessing

After each nightly recording, data from the accelerometer device and from the PSG were downloaded. As the accelerometer and the PSG signals were recorded with two different devices, the clocks of the two signals needed to be synchronized before sleep stages could be compared. To synchronize, time series with IBIs were derived from the PSG and from the accelerometer signal. For the PSG, R-peaks were localized in the ECG signal, by means of a non-linear transformation in combination with first-order Gaussian differentiation [23]. IBIs were derived from the R-peaks by computing the time differences. For the accelerometer signal, the recently developed method for IBI estimation was used [21]. Clock synchronization was finally achieved by using the PSG clock as reference, and by adjusting the rate and offset of the clock of the acceleration signal such that the cross correlation between the two IBI time series was maximized. To this end, we recomputed the IBI sequence from the accelerometer signal $\left(t_{i},IBI_{i}\right),$ by retiming it with offset o and rate r, such that the adjusted IBI sequence became: $\left(o+rt_{i},IBI_{i}\right)$, with i=1…N, $t_{i}$ the localization time of beat i, and $IBI_{i}$ the time difference between beat i+1 and beat i. We then plotted the IBI sequence derived from the ECG signal of the PSG, together with the adjusted IBI sequence derived from the accelerometer, using the times given by the PSG clock on the horizontal axis and the two IBI time series on the vertical axis. In an iterative way, we manually updated o and r until visually an optimal alignment between the time series was achieved, corresponding to maximum cross correlation. As both the PSG system and the accelerometer device used crystal clocks, the rate r was always very close to 1.0.

For the scoring of reference sleep stages, we employed Somnolyzer, an automatic sleep staging method that takes PSG signals as input and combines deep convolutional neural networks with Long Short-Term Memory units to predict sleep stages for each 30-s epoch [24,25]. Somnolyzer was validated in multiple studies against up to 12 different scorers from up to 6 different centers and reached a Cohen’s kappa coefficient of agreement (kappa) versus consensus scoring of 0.79, i.e., levels of agreement comparable to human interrater reliability [26].

### 2.3. Sleep Stage Estimation

**Figure 1:**
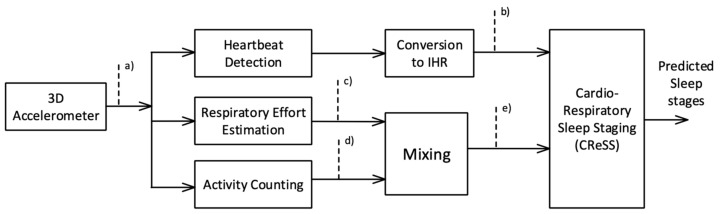
Block diagram of the sleep staging algorithm. Blocks depict algorithmic steps, while arrows carry the signals. The dashed lines with labels refer to the signals in Figure 2. Heartbeats, respiratory effort, and activity counts are derived from the 3D accelerometer signal (**a**). The instantaneous heart rate (IHR, (**b**)) is derived from the heartbeats. The respiratory effort (**c**) and activity counts (**d**) are mixed to obtain a signal (**e**), which combines respiratory effort with body motion. Finally, sleep stages are derived by means of cardio-respiratory sleep staging.

**Figure 2:**
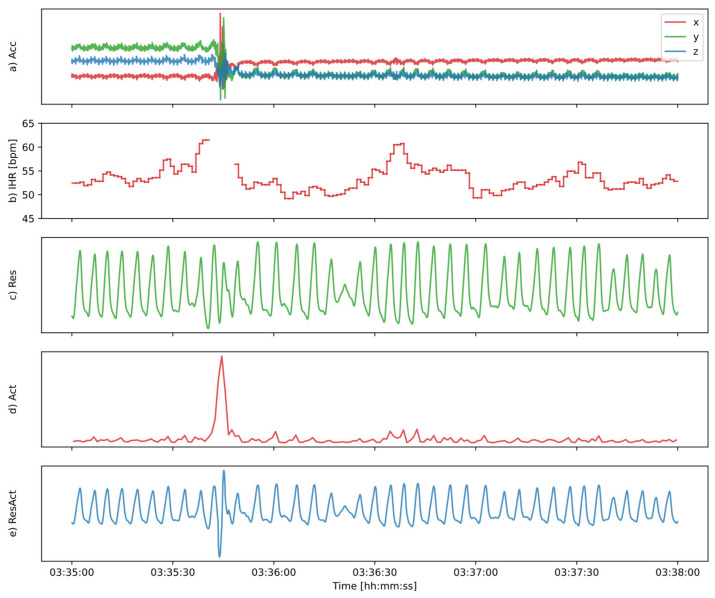
Waveforms when estimating sleep stages from the accelerometer. (**a**) The acceleration components x, y, and z (with arbitrary offsets for better visibility), with respiration as slow variations, heartbeats as small peaks, and body movement at t = 03:35:40. (**b**) The instantaneous heart rate (IHR) in beats per minute, with a gap at the time of motion. (**c**) The estimated respiratory effort (continuing during motion). (**d**) The activity counts, with a high amplitude at the time of motion. (**e**) The respiratory signal, with body movements mixed in.

Sleep stages were estimated using the 3D accelerometer, as outlined in the block diagram in Figure 1. In the first stage, respiratory effort, heartbeats, and activity counts were derived, which were subsequently processed and offered as inputs to the sleep stage classifier. Examples of the waveforms during the various stages of processing are shown in Figure 2.

For heartbeat detection, we used a recently published method [21], which first reduced the dimension of the acceleration signal from 3 to 1 by performing a simple arithmetic addition of the channels. Next, using maximum a posteriori (MAP) estimation, it localized heartbeats and estimated IBIs, such that heartbeat localization times coincided with points of maximum acceleration (the small peaks in Figure 2a), and the IBIs matched local periodicity (the time difference between the small peaks). Instantaneous heart rate (IHR) was then computed based on the IBI to the next beat $IHR_{i}=60/IBI_{i}$. Subsequently, the IHR signal was upsampled, with sample-and-hold, to 10 Hz. As a result, we obtained a signal that was constant in between heartbeat localization times and that potentially changed at heartbeats, see Figure 2b. Using the signal quality self-assessment, which is part of the algorithm [21], estimations during periods of low signal quality were removed, as was the case at the motion artifact in Figure 2a, which resulted in a gap in the IHR in Figure 2b, at t = 03:35:45.

To obtain the respiratory effort signal, we used a previously published method [27] which estimated respiration using the slower variations in the acceleration signal, see Figure 2a. It first filtered and decimated the 3D acceleration components x, y, and z to 10 Hz and then transformed these into an orientation signal, comprising two horizontal components ($h_{1}$, $h_{2}$) and a vertical component (v). The components ($h_{1}$, $h_{2}$) may be interpreted as the coordinates of the bubble in a 2D (planar) spirit level and represent the orientation of the sensor, which follows a pattern that represents the respiratory cycle. A convolutional neural network, which was trained on a separate data set (that was not used in the present study), estimated a respiratory signal that, in the absence of body movements, resembles the signal that is otherwise acquired by a thoracic band, see Figure 2c. 

In respiratory effort signals, measured with recommended sensors such as thoracic belts, body movement artifacts are inevitably present. CReSS was trained on these signals and therefore relies on such artifacts to be present as well. The respiratory effort neural network is very accurate in the estimation of respiration in the absence of movement artifacts, and during periods of movement, it tends to produce a relatively clean signal, unlike traditional sensors. An example is the body movement artifact at t = 03:35:45 in Figure 2a, where the respiratory effort signal in Figure 2c continues without much disturbance. To avoid having to retrain CReSS, we aimed at mimicking body movement artifacts, such that the respiratory effort signal better resembled traditional sensors, by estimating. Towards this end we estimated gross body movements, quantified with so-called “activity counts”. The body movements were estimated each second, using the acceleration signal over the last second, by removing the mean for each of the components (x, y and z), taking the absolute value, and summing over all samples and components: $Act=\frac{1}{N}\sum_{i=1}^{N}\left|x_{i}-\bar{x}\right|+\frac{1}{N}\sum_{i=1}^{N}\left|y_{i}-\bar{y}\right|+\frac{1}{N}\sum_{i=1}^{N}\left|z_{i}-\bar{z}\right|$, with Act the activity counts, and N the number of samples per second. The resulting signal in Figure 2d was scaled and upsampled to 10 Hz, after which it was mixed with the respiratory signal to obtain an actigraphy-modified respiratory effort signal ResAct. Mixing was performed by multiplying the respiratory effort signal Res by the activity counts Act, after offsetting the latter by 1.0 to avoid attenuation: $ResAct=\left(1.0+Act\right)*Res$, with Res, Act, and ResAct, as in Figure 2c–e.

Sleep stage estimation was finally achieved with CReSS, presented in earlier work [13]. CReSS was designed to be device-agnostic and work with respiratory signals (airflow, respiratory effort, or a combination of the two) and instantaneous heart rate inputs. CReSS uses different branches of deep convolutional networks to extract high-level features from both cardiac and respiratory signals. The feature vectors from the individual branches are merged to create a 192-element high-level cardiorespiratory feature vector for each 30-s epoch. Finally, 3 layers of bidirectional Long Short-Term Memory units introduce global context from the entire recording and a fully connected dense layer assigns probabilities for the stages Wake, REM, Light Sleep (N1+N2), and Deep Sleep (N3), from which the stage with the highest probability is then selected.

### 2.4. Performance Evaluation

Sleep stage estimation was evaluated in terms of epoch-per-epoch agreement with the reference sleep stages, using accuracy and Cohen’s kappa coefficient of agreement. We computed agreement for classification of four classes (Wake/N1+N2/N3/REM), three classes (Wake/NREM/REM, obtained by merging N1+N2 and N3 in a single non-REM or NREM class), and a binary classification of each of the four classes against the rest (Wake vs. combined N1+N2, N3 and REM, etc.). In addition, we computed the confusion matrix, from which we derived sensitivity, specificity, and positive predictive value (PPV) for the binary classification tasks. 

Furthermore, we evaluated the estimations of sleep metrics by calculating the total sleep time, sleep efficiency, sleep latency, and time in each stage from the sleep stages estimated from the accelerometer and from the PSG. 

Finally, we evaluated the impact of different factors on sleep staging performance by assessing sleep stage agreement, using a Mann–Whitney U test, across gender and the presence of each sleep disorder (see Table S2 of the Supplemental Materials). For age and BMI, we performed a Spearman’s rank correlation with kappa and found correlations of −0.195 for Age (*p* < 0.001) and of −0.053 for BMI (*p* = 0.346). We used a Benjamini–Hochberg correction, with an acceptable false discovery rate of 5% to prevent false discoveries, and found that the correlation with Age was significant.

## 3. Results

PSG and accelerometer data were collected for a total of 323 participants (Table 1). Sleep disorders were diagnosed following standard clinical practice in each study, according to the criteria of the international classification of sleep disorders (ICSD-3) [28]. The most prevalent sleep disorders were sleep-disordered breathing (SDB, N = 175, 54%) and insomnia (N = 76, 24%). Table S1 in the Supplemental Materials provides a detailed overview of the prevalence of sleep disorders among the participants.

Sleep stages were derived from PSG and from the accelerometer. Table 2 gives the performance of sleep stage classification for each task, with the inter-quartile ranges (if not normally distributed) or the standard deviations (if normally distributed). The sample statistics are calculated across the performance per participant (N = 323). We found a median four-class (Wake/N1+N2/N3/REM) kappa of 0.678 and a median three-class (Wake/NREM/REM) κ of 0.745. As expected, the three-class kappa was higher, because confusion between N1+N2 and N3 is removed when merging these two into the (single) NREM class. For the binary classification task Wake versus Sleep (Wake detection), we found a median κ of 0.723, a median sensitivity of 78.7%, and a median specificity of 96.6%. This is in line with the last row of the confusion matrix (Table 3), which shows that 20.9% of the Wake epochs are classified as N1+N2, 0.4% as N3, and 2.8% as REM. Confusion between REM, Wake, and N3 is all < 5%; confusion > 5% is always in relation to N1+N2. 

Note that Table 3 gives numbers over all epochs of all participants, while Table 2 gives the means and quartiles over performance statistics computed for each participant individually. Therefore, the sensitivities, specificities, and PPV values in Table 2 are not exactly equal to the ones that may be derived from Table 3.

**Figure 3:**
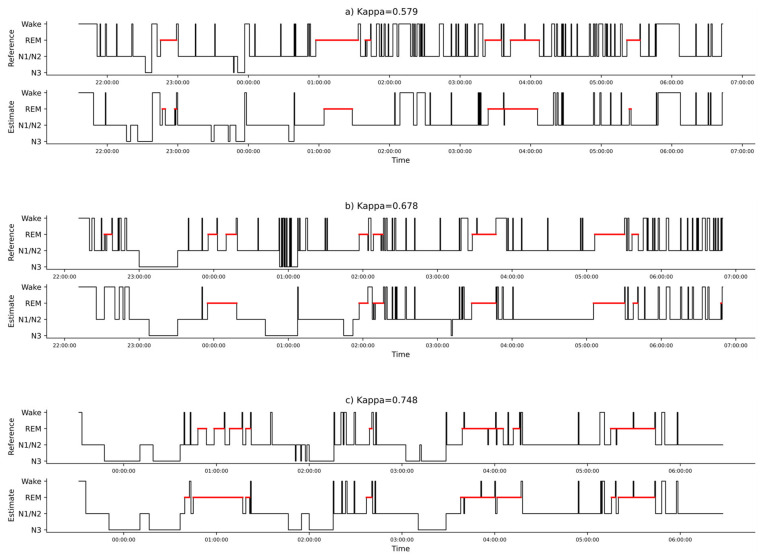
Reference and estimated hypnograms for three recordings: (**a**) near the first quantile (κ = 0.579), (**b**) near the median (κ = 0.678), and (**c**) near the third quantile (κ = 0.748).

Figure 3 illustrates three examples of sleep stage classifications, chosen as the recordings with the four-class κ closest to the first quartile (κ = 0.579), to the median (κ = 0.678), and to the third quartile (κ = 0.748). Furthermore, and beyond the κ metric, intervals of occurrence often match; if we define a REM interval as the longest possible interval in which REM occurs, without it being interrupted by more than M epochs, then we find four REM intervals in the reference in Figure 3a (M = 20). We also find four REM intervals in the estimation in Figure 3a, and we find that these intervals overlap with the reference. Similarly, we find four N3 intervals in the reference of Figure 3c, with four overlapping N3 intervals in the estimation. However, occurrences do not always match, as may be found in Figure 3b, where four REM intervals match, but where the first REM interval in the reference (t ≈ 22:30) has no matching REM interval in the estimation. 

Table 4 gives the performance of the estimation of sleep metrics, with the interquartile ranges for the reference value and for the estimation error in between curly brackets. Furthermore, the 95% limits of agreement are given in the rightmost column. The total sleep time was estimated, with a median error of 2.5 min, first and third quartiles at −9.5 and +17.0 min, and 95% limits of agreement at −51.0 and +107.7 min. The third quartile and upper limit of agreement having higher magnitudes than the first quartile and lower limit of agreement is in line with the confusion matrix in Table 3. Relatively many Wake epochs were scored as Sleep (20.9% N1+N2, 0.4% N3, 2.8% REM, bottom row), while relatively few Sleep epochs were scored as Wake (5.9% N1+N2, 0.4% N3, 2.1% REM, rightmost column). For sleep efficiency, we found a median error of 0.5%, with first and third quartiles at −2.0 and +3.0%, and 95% limits of agreement at −10.9 and +21.1%.

Regarding the influence of factors on the epoch-by-epoch agreement (κ), and after application of the Benjamini–Hochberg correction for false discoveries (FDR < 0.05), we found a significant influence of age. Spearman’s rank correlation was −0.195, hence yielding better performance for younger participants. We did not find significant influences of the presence of SDB and insomnia, which were the two most prevalent disorders in our study (54.2% and 23.5%).

## 4. Discussion

In this study, we evaluated the performance of sleep staging from a chest-worn accelerometer among a group of 323 participants with various sleep disorders. Making use of existing methods for cardio-respiratory sleep staging in combination with estimation methods for cardiac and respiratory characteristics, we evaluated the performance with studies in two sleep centers and compared against reference scoring from PSG. For sleep stage classification we found a kappa for four-class scoring of 0.678, and a kappa of 0.723 for Wake (versus Sleep) detection, both indicative of “substantial agreement” with PSG [29]. 

To put the performance obtained in our study in perspective, in a systematic review on methods for cardiorespiratory sleep staging, one can find kappa values ranging from 0.55 to 0.76 (median = 0.55), and accuracies ranging from 58% to 90% (median = 78%) [12]. It should be noted, however, that there are many differences between the reviewed studies and our work, such as the study populations, the number of sleep stages (the number of classes), and the way the cardiorespiratory signals were obtained (PSG versus “surrogate” signals). The algorithm that we used in the present study, CReSS, was validated earlier among a population of 296 participants with a wide range of SDB severity. Using IHR derived from ECG or finger-worn PPG, respiratory effort from thoracic belts, and airflow, it achieved a kappa of 0.643, without significant differences between different SDB severities [13]. These results are like those obtained in the present study, confirming the suitability of deriving cardiorespiratory inputs from an accelerometer instead.

All confusions > 5% occurred between N1+N2 on the one side, and Wake, REM, and N3 on the other (see Table 3). The CReSS validation study [13] provides confusion matrices for classification using airflow as input, but not specifically with respiratory effort in the absence of airflow (as is the case here). However, the study reveals that the highest confusion occurs between N1+N2 and the remaining sleep stages Wake, REM, and N3, which aligns with our findings. A recent review of cardiorespiratory sleep staging methods [12] unfortunately does not describe possible sources of confusion. It remains a topic for further research whether this is inherent to the intrinsic limitations of cardiorespiratory sleep staging and the information is only present in the neurological signals, or if the confusion is a specific limitation of the chosen algorithm (CReSS).

Relatively many Wake epochs (20.9%, Table 3) were scored as light sleep (N1+N2). This elevated the upper bound on the 95% limits of agreement for sleep efficiency (21.1%, Table 4). Retraining the cardiorespiratory sleep staging on the accelerometer data, possibly targeting the accuracy of the total sleep time, may improve this aspect.

When developing a new sleep staging method, an important choice is how to obtain the reference ground-truth sleep stages. Most often, these are obtained by a single technician scoring PSG, according to the rules of the AASM [1]. To increase scoring reliability, the AASM has described the rules in detail and regularly issues updates. Despite these efforts, human interrater reliability remains imperfect, with kappa values reaching about 0.76 [30], and with the percentage of epochs where all scorers fully agree progressively declining with an increasing number of scorers, down to 25% with 15 scorers or more [25]. This effect can be worse if the data are collected at different centers with staff that have different levels of experience. 

One way to counteract this effect is to obtain a ground truth by having the data scored by multiple technicians, from accredited sleep centers, all trained to the same version of the rules. The consensus of these scorers can then be considered a “gold-standard” reference. However, the associated cost and organizational effort make this approach often not feasible, especially for larger datasets. In the present study, we addressed this issue by employing an automatic scoring method trained on the consensus scorings of multiple experts and widely validated in different external cohorts. Somnolyzer is such a method, representing the consensus opinion of multiple expert scorers and thereby reducing the epistemic uncertainty associated with inter-institutional and scorer-dependent variability [25]. Somnolyzer has been cleared by the FDA as a tool for clinical and pharmacological studies and has been certified by the AASM for sleep staging through their Autoscoring Certification Program [31], a process during which a panel of nine sleep technicians and two physicians assess performance by conducting a detailed review of 100 PSG recordings.

A possible limitation of the approach is that it may overestimate the performance of the accelerometer-based sleep staging method, as there is partial overlap between the datasets on which Somnolyzer and CReSS have been trained. However, we believe this bias is limited, as shown by the hold-out validation of both algorithms in completely independent, external validation datasets [26].

Although we use an accelerometer, our method differs from wrist actigraphy. Wrist actigraphy solely relies on the measurement of body movements, which are used for the estimation of “rough” parameters like the sleep–wake rhythm or the total time in bed. Wrist actigraphy detects Wake with high sensitivity, but with low specificity, as wakefulness without motion is easily confused with sleep. A study on the validation of actigraphy showed a sensitivity of 93% to 99% and a specificity of 37% to 62% in 25 healthy young adults [32]. Our method also uses body movements, but additionally uses small-amplitude accelerations to estimate cardio-respiratory activity. For comparison, our method detected sleep with a sensitivity of 96.6% and a specificity of 78.7% among a large SDB population with a wide range of ages. For the related sensing modality of ballistocardiography (where cardiac activity, and possibly respiration, are measured through the structure on which the body resides), several studies were published. For a small numbers of healthy participants, by using bed sensors, three-class kappa values were reported of 0.44 (eighteen participants) [33], 0.55 (seventeen participants) [34], 0.63 (seventeen participants) [35], and 0.74 (five participants) [36]. The present method reached a three-class kappa of 0.745 among a diverse and disordered group of 323 participants.

We found that sleep staging performance was slightly better in younger individuals (Spearman’s rank correlation coefficient = −0.19). This might be explained by younger participants having more pronounced heart rate variability, as is known from literature [37]. Notably, we found no significant influences of the presence of SDB and insomnia, which were the most prevalent sleep disorders in our study. The consistency of performance due to the presence of SDB is in line with an evaluation of CReSS among a cohort with a large range of SDB severities [13].

There are multiple advantages to performing sleep staging using a chest-worn accelerometer. The technology can be integrated into devices that are already worn on the chest during the night. Examples are devices for positional therapy for OSA (also known as sleep position trainers) and patches for extended Holter monitoring. Unlike most other technologies for sleep staging, our method only needs a single point of mechanical contact with the human body, without the need for direct skin contact, which avoids the need for skin preparation (like shaving), avoids skin irritation, and makes the technology robust against contact deterioration due to, for example, the drying-out of electrodes.

A limitation of this study was that participants with a known history of heart rhythm disorders were excluded. As atrial fibrillation is often comorbid with obstructive sleep apnea [38], the use of our method among populations with suspected obstructive sleep apnea may be problematic. Correlations between sleep stages and heart rate variability may be masked by the rhythm disorder. Further research may reveal whether this is the case. Another limitation was that the accelerometer was mounted by clinical staff, at a defined location on the thoracic band. The influence of self-attachment, as well as the influence of different sensor locations on the body as a result, could be investigated in a follow-up study. Finally, CReSS, which was trained on cardio-respiratory signals derived from PSG, was taken as-is. Cardiorespiratory features derived from PSG may differ from those derived from an accelerometer, as was shown in our earlier work on the estimation of respiratory effort from an accelerometer [27]. Therefore, further research may be conducted in adapting the sleep staging to the signals from the accelerometer, for example by means of transfer learning, which may improve the performance further. 

## 5. Conclusions

We have shown that cardio-respiratory information obtained by a chest-worn accelerometer can be used to assess sleep stages in a sleep-disordered population with diversity in age, BMI, and disease severity. Performance was not significantly influenced by the presence of insomnia and SDB. This provides a new low-obtrusive method for the longitudinal monitoring of sleep in a variety of applications, such as devices for positional OSA therapy and patches for patient monitoring.

## Figures and Tables

**Table 1:** Study demographics.

Dataset	N	Female	Age ^1^ (Years)	BMI ^1^ (kg/m^2^)
KH	195	77 (39%)	52 ± 17 [13–83]	26 ± 4 [19–37]
OLVG	128	41 (32%)	46 ± 13 [21–78]	28 ± 5 [19–47]
KH + OLVG	323	118 (37%)	50 ± 15 [13–83]	28 ± 4 [19–47]

^1^ Entries are given with standard deviation (±) and ranges ([low–high]).

**Table 2:** Results for sleep stage agreement.

Task	Cohen’s Kappa ^3^	Accuracy ^3^ (%)	Sensitivity ^3^ (%)	Specificity ^3^ (%)	PPV ^1,3^ (%)
Wake/N1+N2 /N3/REM	0.678 {0.577, 0.749}	80.8 {75.6, 84.6}			
Wake/NREM /REM	0.745 {0.650, 0.808}	87.5 {83.1, 90.6}			
N1+N2 ^2^	0.617 {0.510, 0.699}	81.8 {77.2, 85.3}	85.4 {80.2, 90.4}	79.1 {70.0, 85.1}	82.6 {76.8, 88.7}
N3 ^2^	0.645 {0.363, 0.769}	93.6 {91.1, 95.7}	66.3 ±27.4	97.6 {94.5, 99.4}	65.3 ±32.3
REM ^2^	0.779 {0.641, 0.861}	94.8 {92.1, 96.7}	76.8 ±21.1	97.9 {95.7, 99.1}	78.0 ±23.9
Wake ^2^ (vs. Sleep)	0.723 {0.609, 0.804}	93.3 {89.7, 95.4}	78.7 {66.2, 88.3}	96.6 {93.9, 98.1}	81.1 {68.4, 90.0}

^1^ PPV = Positive Predictive Value. ^2^ Binary classification tasks were evaluated in a one vs. rest strategy, where one single class (e.g., Wake, REM, etc.) was considered the “positive” class, and the remaining were aggregated in a single “negative” class. ^3^ Entries are given as mean with standard deviation (±) if normally distributed, or else as median with first quartile and third quartile ({Q1, Q3}).

**Table 3:** Confusion matrix ^1^.

Estimate → Reference ↓	N1+N2	N3	REM	Wake
**N1+N2**	144,578 (84.2%)	10,334 (6.0%)	6558 (3.8%)	10,150 (5.9%)
**N3**	10,943 (33.6%)	21,238 (65.2%)	271 (0.8%)	126 (0.4%)
**REM**	9744 (20.7%)	45 (0.1%)	36,395 (77.1%)	1001 (2.1%)
**Wake**	14,009 (20.9%)	293 (0.4%)	1865 (2.8%)	50,898 (75.9%)

^1^ Confusion is over all epochs and all participants (N = 318,448). Percentages are the number of epochs of the predicted class relative to those of the reference class.

**Table 4:** Sleep statistics.

Statistic	PSG ^3^	Error ^1,3^	95% LoA ^2^
Total sleep time (min)	412 {338, 454}	2.5 {−9.5, 17.0}	−51.0, 107.7
Sleep latency (min)	16 {6, 37}	1.5 {−2.5, 5.5}	−42.8, 27.9
Wake after sleep onset (min)	60 {32, 106}	−3.0 {−16.8, 8.0}	−101.5, 45.0
Stage R latency (min)	120 {86, 170}	0.5 {−1.5, 3.0}	−140.8, 131.2
Sleep efficiency (%)	83 {72, 90}	0.5 {−2.0, 3.5}	−10.9, 21.1
Time in Wake (min)	84 {48, 138}	−2.5 {−17.0, 9.5}	−107.8, 50.9
Time in REM (min)	76 {48, 97}	−3.5 {−15.5, 7.5}	−53.0, 41.3
Time in N1+N2 (min)	274 {229, 308}	10.0 {−16.8, 36.0}	−72.5, 114.0
Time in N3 (min)	50 {25, 71}	−1.5 {−19.5, 16.8}	−64.5, 61.9
Percentage of TST in Wake (%)	20 {11, 40}	−0.6 {−5.9, 3.2}	−55.7, 21.9
Percentage of TST in REM (%)	19 {15, 24}	−1.2 {−4.2, 1.9}	−13.3, 10.0
Percentage of TST in N1+N2 (%)	67 {61, 74}	2.1 {−4.3, 7.3}	−18.1, 23.2
Percentage of TST in N3 (%)	13 {7, 18}	−0.6 {−5.6, 4.5}	−17.1, 17.2

^1^ The error is computed as the value derived from the accelerometer minus the value derived from the scoring of PSG. ^2^ The 95% limits of agreement (LoA) refer to the error. ^3^ Each entry contains the median followed by the first and third quantile in between curly brackets.

## Data Availability

The datasets presented in this article are not readily available because they are owned by a third party, and the terms of use prevent public distribution.

## Supplementary Materials

The following supporting information can be downloaded at: https://www.mdpi.com/article/10.3390/s24175717/s1, Table S1. Prevalence of Sleep Disorders in the dataset (N = 323). Table S2. Influence of factors on the epoch-by-epoch agreement (kappa).
