# Supplementary material for: Overnight Sleep Staging Using Chest-Worn Accelerometry
Source: Sensors (Basel). 2024 Sep 2;24(17):5717. doi: 10.3390/s24175717 (PMC11398147; doi:10.3390/s24175717)
Supplement: Supplementary file 1 [file sensors-24-05717-s001.zip › sensors-3082269-supplementary.pdf]

Supplemental materials to "Overnight sleep staging with chest-worn accelerometry"

Table S1. Prevalence of Sleep Disorders in the dataset (N=323)

	Total prevalence	Single Primary Disorder
**SDB**	175 (54.2%)	113 (35.0%)
**Insomnia**	76 (23.5%)	25 (7.7%)
**REM Parasomnia**	41(12.7%)	12 (3.7%)
**Movement disorder**	36 (11.1%)	5 (1.5%)
**Non-REM Parasomnia**	26 (8.0%)	13 (4.0%)
**Hypersomnia**	14 (4.3%)	5 (1.5%)
**Behavioral**	8 (2.5%)	3 (0.9%)
**Circadian disorder**	7 (2.2%)	2 (0.6%)
**None**	4 (1.2%)	3 (0.9%)

The first column shows the number of participants with the respective diagnosis, grouped according to major ICSD-2 categories (with Parasomnia split in REM and Non-REM subcategories). The second column shows the number of participants for whom the respective diagnosis was the single primary sleep disorder.

Table S2. Influence of factors on the epoch-by-epoch agreement (kappa)

Factor	Median kappa in absence	Median kappa in presence	Difference	p-value
**REM Parasomnia**	**0.682**	**0.587**	**0.096**	**0.002**
**Non-REM Parasomnia**	**0.674**	**0.755**	**-0.081**	**0.008**
Circadian disorder	0.675	0.780	-0.105	0.035
SDB	0.682	0.671	0.011	0.054
Behavioral disorder	0.674	0.720	-0.046	0.129
Hypersomnia	0.680	0.597	0.083	0.132
Movement disorder	0.680	0.651	0.029	0.180
Insomnia	0.682	0.668	0.014	0.476
Gender	0.704 (female)	0.660 (male)	0.043	0.022

Significant factors (after Benjamini-Hochberg correction with an acceptable false discovery rate of 5%) are shown in bold.
